# BRD4‐IRF1 axis regulates chemoradiotherapy‐induced PD‐L1 expression and immune evasion in non‐small cell lung cancer

**DOI:** 10.1002/ctm2.718

**Published:** 2022-01-26

**Authors:** Jian Wang, Yingzhuo Xu, Xinrui Rao, Ruiguang Zhang, Jing Tang, Dan Zhang, Xiaohua Jie, Kuikui Zhu, Xu Wang, Yunhong Xu, Sheng Zhang, Xiaorong Dong, Tao Zhang, Kunyu Yang, Shuangbing Xu, Rui Meng, Gang Wu

**Affiliations:** ^1^ Cancer Center, Union Hospital Tongji Medical College, Huazhong University of Science and Technology Wuhan 430022 China

**Keywords:** BRD4, cisplatin, non‐small cell lung cancer, PD‐L1, radiotherapy

## Abstract

**Background:**

Chemoradiotherapy‐induced PD‐L1 upregulation leads to therapeutic resistance and treatment failure. The PD‐1/PD‐L1 blocking antibodies sensitize cancers to chemoradiotherapy by blocking extracellular PD‐1 and PD‐L1 binding without affecting the oncogenic function of intracellular PD‐L1. Reversing the chemoradiation‐induced PD‐L1 expression could provide a new strategy to achieve a greater anti‐tumour effect of chemoradiotherapy. Here, we aimed to identify candidate small molecular inhibitors that might boost the anti‐tumour immunity of chemoradiotherapy by decreasing treatment‐induced PD‐L1 expression in non‐small cell lung cancer (NSCLC).

**Methods:**

A drug array was used to recognize compounds that can suppress the cisplatin‐induced and radiation‐induced PD‐L1 expression in NSCLC via the flow cytometry‐based assay. We examined whether and how targeting bromodomain containing 4 (BRD4) inhibits chemoradiation‐induced PD‐L1 expression and evaluated the effect of BRD4 inhibition and chemoradiation combination in vivo.

**Results:**

BRD4 inhibitors JQ1 and ARV‐771 were identified as the most promising drugs both in the cisplatin and radiation screening projects in two NSCLC cell lines. Targeting BRD4 was supposed to block chemoradiotherapy inducible PD‐L1 expression by disrupting the recruitment of BRD4‐IRF1 complex to PD‐L1 promoter. A positive correlation between BRD4 and PD‐L1 expression was observed in human NSCLC tissues. Moreover, BRD4 inhibition synergized with chemoradiotherapy and PD‐1 blockade to show a robust anti‐tumour immunity dependent on CD8+ T cell through limiting chemoradiation‐induced tumour cell surface PD‐L1 upregulation in vivo. Notably, the BRD4‐targeted combinatory treatments did not show increased toxicities.

**Conclusion:**

The data showed that BRD4‐targeted therapy synergized with chemoradiotherapy and anti‐PD‐1 antibody by boosting anti‐tumour immunity in NSCLC.

## Graphical abstract

(1) The flow cytometry‐based drug screening experiments showed that bromodomain Containing 4 (BRD4) inhibitors can simultaneously reverse the expression of PD‐L1 in NSCLC tumour cells induced by radiotherapy and cisplatin treatment.

(2) Radiotherapy and chemotherapy up‐regulating PD‐L1 expression via the BRD4/IRF1 axis in NSCLC.

(3) Targeting BRD4 boosted anti‐tumour immunity of chemoradiotherapy dependent on CD8+ T cells in vivo.

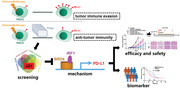

## 1 BACKGROUND

Non‐small cell lung cancer (NSCLC) is the major form of primary lung cancer^1^ and one of the leading causes of cancer‐related death worldwide.^2^ In the management of NSCLC, platinum‐based chemotherapy and radiotherapy have been extensively used when surgical excision is not an option. Concurrent chemoradiotherapy is presently the standard of care for these patients present with stage III disease,^3^ which accounting for approximately 30% of NSCLC patients.^4^ Despite the tremendous efforts and progress in lung cancer research, the long‐term survival of NSCLC patients remains unsatisfactory. Therefore, a better understanding of tumour resistance to chemoradiotherapy and a better solution to overcome resistance are in great need.

Recently, checkpoint blockade immunotherapies targeting the programmed cell death‐1/programmed death ligand 1 (PD‐1/PD‐L1) have intensively been studied and provided clinical activity in varieties of malignancies, including NSCLC.^5^ PD‐L1 (encoded by *CD274*) is frequently overexpressed in various human cancer and expressed in 19.6%–65.3% of NSCLC reportedly.^6^ The interactions between the PD‐1 receptor on T cells and its ligand PD‐L1 on tumour cells induce T cell exhaustion,^7^ and antibodies targeting PD‐1/PD‐L1 signalling can boost the endogenous anti‐tumour immunity.^8^ However, the clinical benefit of immune checkpoint therapy alone is limited by the relatively low response rate at ∼20% in NSCLC and adverse events.^8^ Recent advances indicate that chemoradiotherapy induces anti‐tumoural immunity by releasing tumour‐associated antigens and enhancing major histocompatibility complex (MHC) class I expression on tumour cells.^9^, ^10^, ^11^ But it has also simultaneously led to multifaceted immunosuppressive effects including up‐regulating tumour PD‐L1 expression.^12^ A recent study showed that radiation and deoxyribonucleic acid (DNA)‐damaging drugs‐induced DNA double‐strand break repair enhanced PD‐L1 expression dependent on interferon regulatory factor 1 (IRF1).^13^ Cisplatin is also reported to increase PD‐L1 expression via the cGAS/STING pathway.^14^ In addition to its role as an immune checkpoint, PD‐L1 has intrinsic oncogenic functions that facilitate resistance to radiation or chemotherapy via regulating DNA damage response as an ribonucleic acid (RNA) binding protein.^15^, ^16^ Therefore, targeting chemoradiotherapy‐induced PD‐L1 expression has become a promising approach to improve the clinical benefits of conventional therapy including radiation and cisplatin.^11^, ^17^

The bromo‐ and extra‐terminal domain (BET) family (BRD2, BRD3, BRD4) of epigenetic ‘reader’ proteins recognize and bind competitively acetylated lysine residues on histones and other cellular proteins to facilitate the recruitment of transcriptional factors, which shows their role as important transcriptional regulators.^18^, ^19^ BRD4 inhibitors, JQ1 and ARV771, have shown a wide range of anti‐tumour activity in different types of cancer with manageable toxicity.^19^, ^20^, ^21^ Notably, the latest progress demonstrated that JQ1 modulates tumour immune microenvironment (TIME) by suppressing constitutively PD‐L1 expression and interferon gamma (IFN‐γ)‐stimulated PD‐L1 expression in tumours,^22^, ^23^, ^24^, ^25^ eliciting tumour immunogenicity,^26^ improving activity of T cell^24^, ^27^ and natural killer (NK) cell.^28^ Therefore, BET inhibitor‐based combinations limit tumour progress by modulating anti‐tumour immunity and potentially attenuate resistance to immunotherapy.^27^, ^29^

Although several studies successfully identified the mediators of constitutively PD‐L1 expression^30^ and IFN‐γ‐stimulated PD‐L1 expression^31^ in melanoma. However, there are rare screen projects to investigate the unique regulator of therapy‐induced PD‐L1 expression. Moreover, clinically approved antibodies sensitize cancers to chemoradiotherapy by blocking extracellular PD‐1 and PD‐L1 binding^32^ without affecting the oncogenic function of intracellular PD‐L1.^16^ Suppressing the radiation‐induced and cisplatin‐induced PD‐L1 expression could boost the anti‐tumour effect of chemoradiotherapy. Therefore, we conducted a flow cytometry‐based assay using a drug library to recognize compounds that can suppress both the radiation‐induced and cisplatin‐induced cell surface PD‐L1 expression in two NSCLC cells. Here, we found that targeting BRD4 is one of the most promising methods to boost anti‐tumour immunity in combination with radiation and cisplatin as well as concurrent chemoradiotherapy via prohibiting the treatment‐induced PD‐L1 up‐regulation.

## 2 METHODS

Details of cell lines, reagents, radiation, real‐time quantitative PCR (RT‐qPCR), cell viability assay and apoptosis detection, gene silencing, Western blot, immunoprecipitation (IP), immunofluorescence (IF) and immunohistochemistry, hematoxylin‐eosin staining, database analysis, RNA sequencing (RNA‐seq), cytokine assays, in vitro co‐culture assay, etc. were described in supplementary methods.

### 2.1 CUT&Tag assay

CUT&Tag (Cleavage Under Targets and Tagmentation) analysis was performed with the CUT&Tag kit (Vazyme Biotech),^33^ using specific antibodies for BRD4 (#13440, Cell Signaling Technology), IRF1 (#8478, Cell Signaling Technology).^34^ CUT&Tag‐immunoprecipitated DNA samples were analyzed by RT‐qPCR and normalized to input with an isotype‐matched IgG as a negative control. Specific primers targeting the CD274 promoter were designed according to a published study, and the used primers were ‐103 forward: TGAAAGCTTCCGCCGATTT, ‐53 reverse: TGCCGGGCGTTGGA.^34^

### 2.2 Animal experiments

The animal studies were performed following the NIH Guidelines for the Care and Use of Laboratory Animals and authorized by the Animal Care Committee of Tongji Medical College. An inoculum of 5 × 10^5^ Lewis lung carcinoma cells was injected subcutaneously on the dorsal flank of C57BL/6 mice (5∼6 weeks old, female, Changzhou Cavens Laboratory Animal Co., Ltd) in 100 μl sterile PBS. Mice with palpable tumours were randomized into different groups. Drugs and reagents were stored and diluted according to the manufacturer's instructions and administered as follows: JQ1, intraperitoneally (i.p.), 50 mg/kg^29^; cisplatin, i.p., 5 mg/kg^35^; anti‐PD‐1 antibody (BE0146, BioXCell), i.p., 10 mg/kg^36^; CD8α depletion antibody (2.43, BioXcell), 150 μg per animal and three times weekly.^37^, ^38^ Local radiotherapy in the tumours (8 Gy) was given on three consecutive days.^37^, ^39^ Mice were monitored and weighted, and tumour volume was calculated with the formula: (L*W^2^)/2. Mice were sacrificed when moribund or tumours reach about 1000 mm^3^ for the welfare of animals. At the indicated time and the end of experiments, tissues and serum were collected and prepared for analysis.

### 2.3 Flow cytometry phenotyping

To measure PD­L1 levels on the cell surface of tumour cells treated with vehicle, cisplatin, radiation, JQ1, IFN‐γ (20 ng/ml,^24^ PeproTech), or the combinations for the different time periods were trypsinized and harvested for staining with streptavidin‐phycoerythrin (PE)‐conjugated anti‐human PD‐L1(329706, Biolegend). Tumour tissues were collected, cut into small pieces, digested with a mixture of collagenase, DNase and hyaluronidase for preparing single‐cell suspension and then treated with Cell Stimulation Cocktail (00‐4975‐03, eBioscience) as instructed. Cells were washed, re‐suspended in fluorescence‐activated cell sorting buffer at 4°C and then stained with fluorescent‐conjugated antibodies and appropriate isotype controls for multicolor flow cytometry. For cell surface staining, the following antibodies were used: fluorescein isothiocyanate (FITC) anti‐mouse CD3 (100204, Biolegend), PE/Cy7 anti‐mouse cluster of differentiation 4 (CD4) (100422, Biolegend), APC anti‐mouse CD8 (100712, Biolegend), Brilliant Violet 605 anti‐mouse NK1.1 (108739, Biolegend) and PE anti‐mouse PD‐L1(124308, Biolegend). The collected live single cells were fixed and treated with permeabilization buffer before intracellular staining with Brilliant Violet 421 anti‐mouse IFN‐γ (505830, Biolegend).

### 2.4 Statistical analysis

Data were shown as mean ± standard deviation unless indicated otherwise. For comparing two groups, the unpaired two‐tailed Student's test was used. For multiple groups, the one‐way analysis of variance was used. For survival analysis, the Kaplan–Meier method and the Gehan–Breslow‐Wilcoxon test were performed. Pearson's correlation test was used for correlation analysis, and Pearson's correlation coefficients (R) were calculated. A two‐tailed test with *p* < .05 was statistically significant.

## 3 RESULTS

### 3.1 BRD4 inhibition was identified to suppress radiation‐induced and cisplatin‐caused PD‐L1 up‐regulation in NSCLC

**FIGURE 1:**
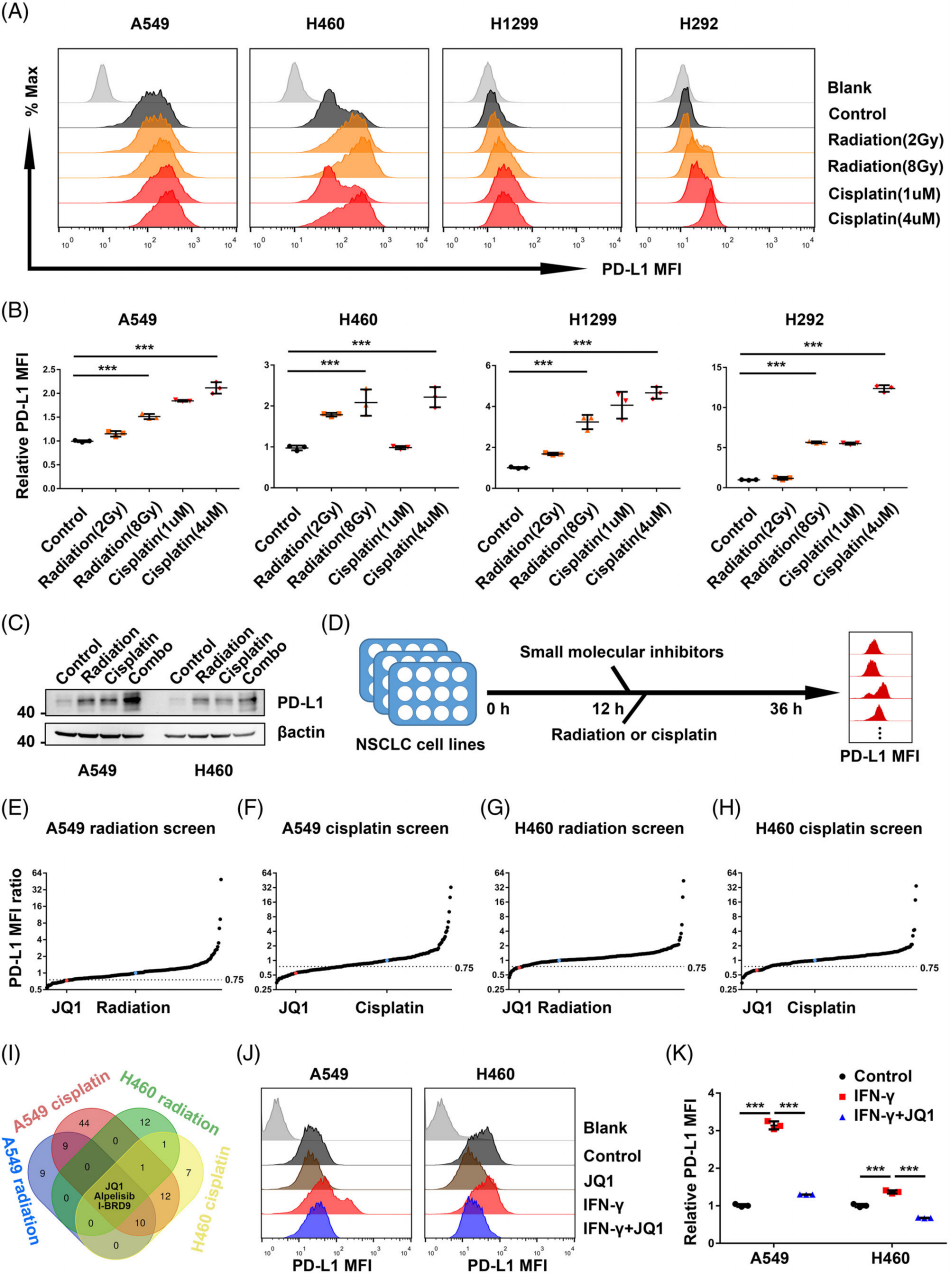
Drug screenings identified JQ1 as an inhibitor of radiation‐induced and cisplatin‐induced programmed death ligand 1 (PD‐L1) up‐regulation in non‐small cell lung cancer (NSCLC). (A) Representative fluorescence‐activated cell sorting (FACS) images and (B) quantitation of cell surface PD‐L1 expression in NSCLC cells treated with the indicated dose of radiation and cisplatin for 48 h (*n* = 3). (C) Western blot analysis of the expression of PD‐L1 and β‐actin in NSCLC cells treated with vehicle, radiation (8 Gy), cisplatin (4 μM) and the combination for 36 h. (D) Schematic diagram of the screening strategy. 2 × 10^5^ A549 and H460 were seeded overnight and treated with radiation (8 Gy) or cisplatin (4 μM) after adding the inhibitors (10 μM) to the culture medium. Following incubation for 24 h, tumour cells were collected for single‐cell suspension and detected surface PD‐L1 expression by flow cytometry. (E) Results of the A549 radiation screen. Mean fluorescence intensity (MFI) ratio = (MFI_(candidate drug + radiation)_ ‐ MFI_isotype_)/(MFI_radiation_ ‐ MFI_isotype_). PD‐L1 MFI ratio values for each inhibitor were plotted to recognize inhibitor with PD‐L1 MFI ratio <.75. (F) Results of the A549 cisplatin screen. Mean fluorescence intensity (MFI) ratio = (MFI_(candidate + cisplatin)_ ‐ MFI_isotype_)/(MFI_cisplatin_ ‐ MFI_isotype_). (G) Results of the H460 radiation screen. (H) Results of the H460 cisplatin screen. (I) Venn diagram of intersecting ‘hits’ from the four indicated inhibitor screens above. (J) Representative FACS images and (K) quantitation of cell surface PD‐L1 expression in NSCLC cells treated with JQ1 (1 μM) with the stimulation of interferon gamma (IFN‐γ) (20 ng/ml) for 48 h (*n* = 3)

To investigate whether conventional therapies including radiation and cisplatin influence the PD‐L1 level of cancer cells, we analyzed the tumour cell surface PD‐L1 level in four NSCLC cell lines by flow cytometry. Similar to published reports,^13^ we confirmed that radiation‐induced PD‐L1 up‐regulation in a dose‐dependent way in A549, H460, H1299 and H292 cells (Figure 1A,B). In line with previous studies,^14^, ^35^ the results showed that cisplatin increased PD‐L1 level on NSCLC cell surface in a dose‐dependent way (Figure 1A,B). Furthermore, a more significant PD‐L1 increase in NSCLC treated with the combination of radiation and cisplatin was observed (Figure 1C). Targeting chemoradiotherapy‐induced PD‐L1 is a promising strategy to augment anti‐tumour immunity and overcome treatment resistance.^40^ Although previous screen projects identified the mediators of constitutively PD‐L1 expression^30^ and IFN‐γ‐stimulated PD‐L1 expression^31^ in melanoma. However, there may be different mechanisms underlying the inducible PD‐L1 expression in cancer cells treated with radiation and cisplatin. Identification of the unique regulators of chemoradiation‐induced PD‐L1 up‐regulation is of great value. Consequently, we conducted the first flow cytometry‐based assay^31^ using a drug array with about two hundred agents to recognize novel inhibitors that could suppress both the radiation‐induced and the cisplatin‐induced cell surface PD‐L1 expression in NSCLC. As present in Figure 1D, NSCLC cells, A549 and H460, were treated with radiation (8 Gy) or cisplatin (4 μM) after adding the inhibitors (10 μM) to the culture medium. Following incubation for 24 h, tumour cell surface PD‐L1 expressions were detected by flow cytometry. In the A549 radiation screen, 31 drugs that caused more than a quarter reduction of cell surface PD‐L1 level in A549 treated with radiation were found (Figure 1E and Table S1). In the A549 cisplatin screen, 79 agents that elicited more than a quarter reduction of cell surface PD‐L1 level in A549 cells treated with cisplatin were discovered (Figure 1F and Table S1). For the H460 radiation screen and cisplatin screen, 17 agents and 34 agents were recognized, respectively (Figure 1G,H, and Table S1). In the overlapping analysis, three ‘hits’ in the radiation and cisplatin screen of both A549 and H460 were BRD4 inhibitor JQ1, PI3KCA inhibitor Alpelisib and BRD9 inhibitor I‐BRD9 (Figure 1I). PI3KCA inhibition could suppress PD‐L1 expression in an induced model of radioresistance in head and neck cancer,^40^ indicating the reliability of the drug screens described above. Because JQ1‐based therapy could remodel the TIME by different mechanisms.^22^, ^24^, ^26^, ^27^, ^28^, ^29^, ^41^, ^42^ Besides, the lower concentration of JQ1 (1 μM) also blocked IFN‐γ‐induced PD‐L1 up‐regulation in A549 and H460 cells (Figure 1J,K). However, the effect of targeting BRD4 on chemoradiotherapy‐induced PD‐L1 expression in NSCLC tumour cells is poorly understood. So, JQ1 was chosen for further analysis in chemoradiotherapy‐provoked PD‐L1 expression.

### 3.2 Targeting BRD4 blocked chemoradiotherapy‐induced PD‐L1 expression

**FIGURE 2:**
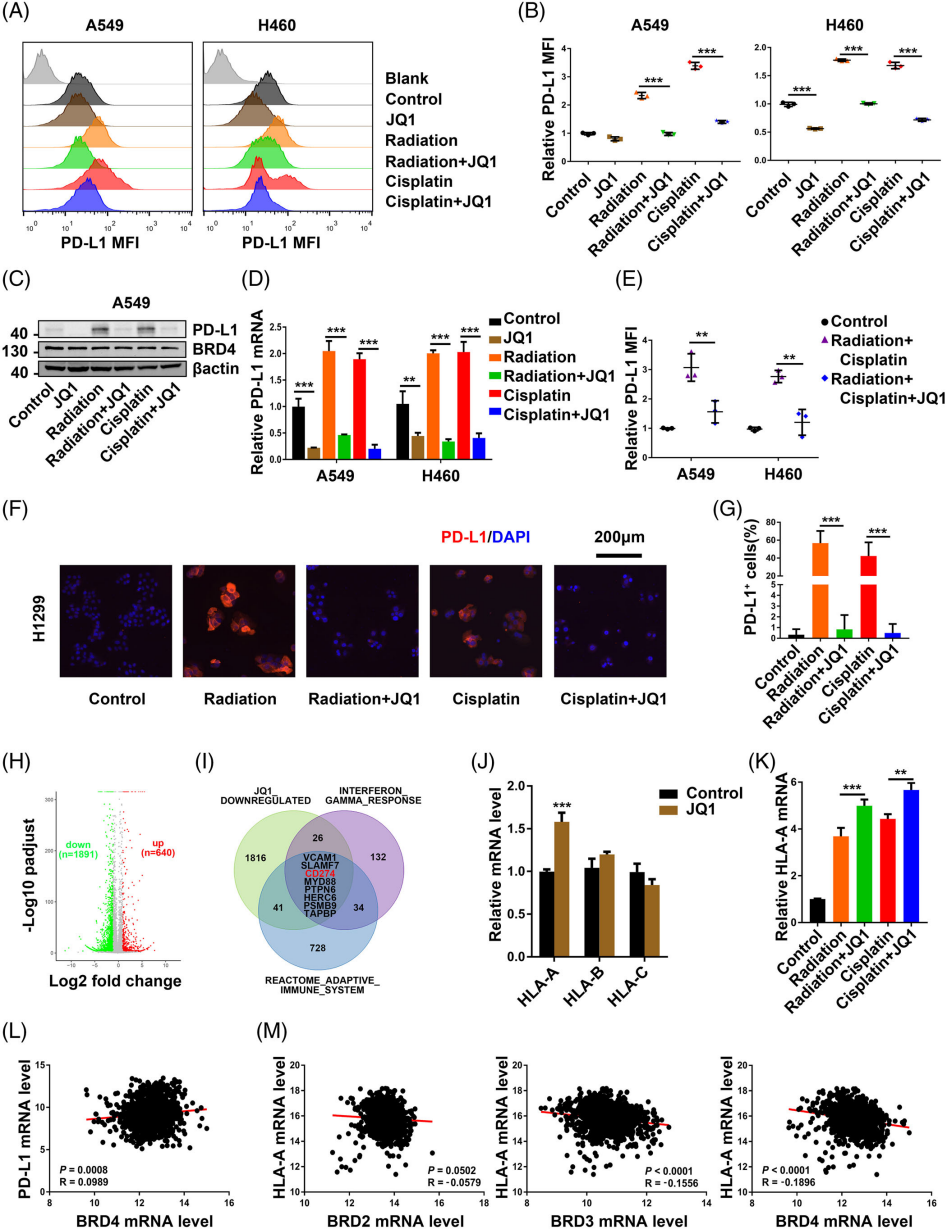
JQ1 blocked chemoradiotherapy‐induced transcription of programmed death ligand 1 (PD‐L1) and increased major histocompatibility complex (MHC)‐1 expression. (A) Representative fluorescence‐activated cell sorting (FACS) images and (B) quantitation of cell surface PD‐L1 expression in non‐small cell lung cancer (NSCLC) cells treated with radiation and cisplatin with or without JQ1 (1 μM) for 48 h (*n* = 3). (C) Western blot analysis of the expression of PD‐L1, BRD4 and β‐actin in A549 treated with radiation (8 Gy) and cisplatin (4 μM) w/o JQ1 (1 μM). (D) Changes of PD‐L1 mRNA in NSCLC cell lines treated with radiation and cisplatin with or without JQ1 (1 μM) for 36 h. Results are normalized to Glyceraldehyde‐3‐phosphate dehydrogenase (GAPDH) (*n* = 3). (E) Changes of cell surface PD‐L1 expression in NSCLC cells treated with the combination of radiation and cisplatin with or without JQ1 (1 μM) for 36 h as in Figure 1B (*n* = 3). (F) Immunofluorescence (IF) staining of PD‐L1 and (G) the percentages of PD‐L1 positive cells in H1299 cells treated with radiation and cisplatin with or without JQ1 (1 μM) for 48 h and counterstained with 4′,6‐diamidino‐2‐phenylindole (DAPI). Scale bar = 200 μm. (H) Volcano plot showing the RNA‐seq results of A549 treated with vehicle or JQ1(2 μM) for 24 h (Table S2). (I) Venn diagram of intersecting genes from the JQ1 down‐regulated genes, interferon gamma (INF‐γ) response genes and adaptive immune system genes (Table S2). (J) Changes of human leucocyte antigen‐A/B/C (HLA‐ABC) mRNA in NSCLC cell lines treated with JQ1 (1 μM) for 36 h. Results are normalized to GAPDH (*n* = 3). (K) Changes of HLA‐A mRNA in NSCLC cell lines treated with radiation and cisplatin with or without JQ1 (1 μM) for 36 h. Results are normalized to GAPDH (*n* = 3). (L) Analysis of correlation between PD‐L1 mRNA and BRD4 mRNA level in NSCLC of The Cancer Genome Atlas (TCGA) dataset. (M) Analysis of correlation between HLA‐A mRNA and BRD2/3/4 mRNA level in NSCLC of TCGA dataset

We performed further validation on JQ1 and found that JQ1 at a lower concentration (1 μM) than that used in the primary screen assays could efficiently inhibit both the radiation‐induced and cisplatin‐induced tumour cell surface PD‐L1 expression in vitro (Figure 2A,B). Similarly, JQ1 decreased both the radiation‐induced and cisplatin‐induced up‐regulation of PD‐L1 protein levels (Figure 2C). JQ1, a well‐characterized inhibitor of BET proteins, may regulate gene expression by disturbing the binding of BET proteins to acetylated lysine residues on proteins and impeding the recruitment of transcriptional factors. Consistent with existing reports,^13^, ^43^ both radiation and cisplatin increased tumour PD‐L1 messenger RNA (mRNA) levels in vitro. Notably, results demonstrated that JQ1 suppressed the radiation‐caused and the cisplatin‐caused up‐regulation of PD‐L1 mRNA in A549 and H460 cells (Figure 2D). This suggests that JQ1 reversed treatment‐provoked PD‐L1 expression at the transcriptional level. Consistently, repression of IFN‐γ‐stimulated PD‐L1 expression by JQ1 also occurred at the transcriptional level in NSCLC (Figure S1A).^24^ Moreover, JQ1 inhibited concurrent chemoradiotherapy‐induced tumour cell surface PD‐L1 expression in NSCLC cells in vitro (Figure 2E). Next, the IF assay demonstrated radiation, and cisplatin enhanced the cytoplasmic membrane labelling of PD‐L1 as compared with control, which could be reversed by JQ1 (Figure 2F,G). In RNA‐seq analysis of A549 cells, JQ1 treatment led to a wide range of gene expression changes, with 1891 genes down‐regulated and 640 genes up‐regulated (Figure 2H). By overlapping analysis, we showed that JQ1 could specifically regulate a class of genes related to immunity including CD274 (encoding PD‐L1) (Figure 2I). We defined the overlapped gene lists as the JQ1 signature and observed a positive correlation between the exhausted T cell signature with PD‐L1 mRNA level and the JQ1 signature in NSCLC (Figure S1B,C).

Additionally, previous studies showed both radiation and cisplatin could regulate MHC‐I (encoded by HLA‐A/B/C in human) expression and enhance the tumour cell antigen presentation.^10^, ^14^ We then evaluated whether JQ1 could affect MHC‐I expression and found that JQ1 increased HLA‐A mRNA expression without significantly changing mRNA levels of HLA‐B and HLA‐C (Figure 2J). JQ1 also synergized with radiation and cisplatin to increase HLA‐A mRNA expression in NSCLC (Figure 2K). A recent report showed that JQ1 preferentially increased HLA‐A mRNA levels in IFN‐γ stimulated prostate cancer.^26^ A slight positive correlation between mRNA levels of PD‐L1 and BRD4 was observed in NSCLC, while no such positive correlations between PD‐L1 and BRD2/3 (Figure 2L, Figure S1D,E). Interestingly, HLA‐A mRNA levels tended to negatively correlate with the mRNA expressions of BRD2, BRD3 and BRD4. There were slight negative correlations between the BRD4 mRNA level and the mRNA expression of HLA‐A and HLA‐B and HLA‐C (Figure 2M, Figure S2F,G). Thus, we identified JQ1 as a suppressor of chemoradiation‐induced PD‐L1 expression.

### 3.3 Chemoradiotherapy enhanced BRD4 to recruit IRF1 to CD274 promoter and up‐regulated PD‐L1 transcription

**FIGURE 3:**
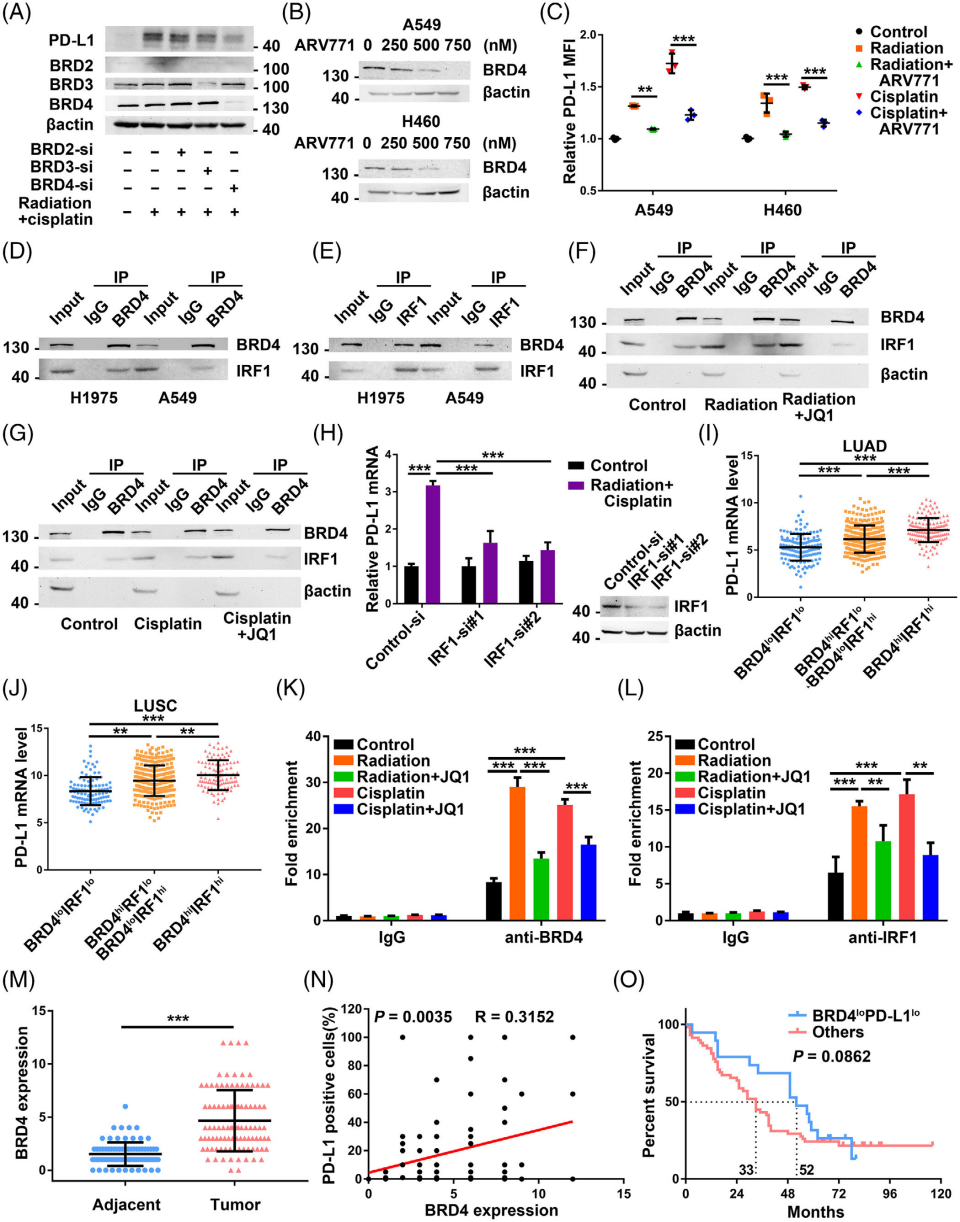
The BRD4‐interferon regulatory factor 1 (IRF1) axis is required for chemoradiotherapy‐mediated programmed death ligand 1 (PD‐L1) up‐regulation. (A) Western blot analysis of the expression of PD‐L1, BRD2/3/4 and β‐actin in A549 transfected with scramble, BRD2/3/4 siRNAs and then treated with the combination of radiation and cisplatin. (B) Western blot analysis of the expression of BRD4 and β‐actin in non‐small cell lung cancer (NSCLC) cells treated with indicated doses of BRD degrader ARV771. (C) Changes of cell surface PD‐L1 expression in NSCLC cells treated with radiation and cisplatin in the presence or absence of ARV771 (.5 μM) (*n* = 3). (D) The NSCLC cells were subjected to co‐immunoprecipitation (IP) assay to detect the interactions between BRD4 and IRF1 using the anti‐BRD4 antibody or isotype‐matched IgG control and then detected by Western blot analysis with the indicated antibodies. (E) The co‐IP assay was performed with the anti‐IRF1 antibody and then detected by Western blot analysis. (F) A549 cells were treated with radiation with or without JQ1 for 24 h, and then subject to the co‐IP assay with the anti‐BRD4 antibody and detected by Western blot analysis with the indicated antibodies. (G) A549 cells were treated with cisplatin with or without JQ1 for 24 h, and then subject to the co‐IP assay with the anti‐BRD4 antibody. (H) PD‐L1 mRNA changes in A549 transfected with the scramble and IRF1 siRNAs and then treated with the combination of radiation and cisplatin. Knockdown efficiencies of IRF1 by the indicated siRNAs were detected by Western blot analysis. (I) PD‐L1 mRNA levels were determined with BRD4 and IRF1 at low (lo) and high (hi) levels in The Cancer Genome Atlas (TCGA) lung adenocarcinoma (LUAD) patients. (J) PD‐L1 mRNA levels were determined with BRD4 and IRF1at low (lo) and high (hi) levels in TCGA lung squamous cell carcinoma (LUSC) patients. (K and L) A549 cells were treated with radiation and cisplatin with or without JQ1 for 24 h, and then subjected to CUT&Tag analysis for the PD‐L1 gene promoter using the indicated antibodies or isotype‐matched IgG control (*n* = 3). (M) BRD4 protein expression in the paired adjacent and tumour tissues in the tissue microarray of NSCLC patients. (N) Analysis of correlation between percentages of PD‐L1 positive cells and BRD4 protein expressions in the tissue microarray of NSCLC patients. (O) Overall survival was determined with BRD4 and PD‐L1 at low and high levels in the tissue microarray of NSCLC patients by using the Kaplan–Meier plotter method

As BET proteins, BRD2/3/4, are major targets of JQ1, we further determined which ones were specifically mediated the repression of chemoradiation‐induced PD‐L1 up‐regulation by JQ1. Interestingly, efficiently BRD4 knockdown resulted in the suppression of chemoradiation‐induced up‐regulation of PD‐L1 protein levels, while no such significant impacts were observed in BRD2 or BRD3 silenced cells (Figure 3A). ARV771, a novel BET PROteolysis TArgeting Chimeric degrader,^44^ has provided an opportunity for exploring the biological functions of BRD4 protein by enhancing BRD4 protein degradation (Figure 3B). As expected, ARV771 inhibited radiation‐induced and cisplatin‐induced tumour cell surface PD‐L1 expression in A549 and H460 cells (Figure 3C). These findings suggest that BRD4 may play important roles in chemoradiation‐provoked PD‐L1 expression.

To find how BRD4 regulates PD‐L1 expression upon chemoradiation, we searched the literature for the transcription factors that might both regulate PD‐L1 transcription and have connections with BRD4. After checking the candidate transcription factors, we chose the IRF1 for further evaluation. First, IRF1 is reported as an important transcription factor to regulate tumour cell PD‐L1 expression by binding to CD274 promoter.^13^, ^45^, ^46^ Then, previous studies showed that BRD4 is another key mediator of both tumour cell constitutive and IFN‐γ‐stimulated CD274 transcription.^24^, ^47^ Moreover, BRD4 could interact with IRF1 to regulate gene transcription in the process of necrosis.^48^ To confirm our hypothesis that BRD4 recruited IRF1 to mediated PD‐L1 expression after radiation and cisplatin treatment, we started with validating the interaction between BRD4 and IRF1 in NSCLC by the co‐IP experiments (Figure 3D,E). Next, results showed that both radiation and cisplatin may promote the association of BRD4 with IRF1 while JQ1 partially disrupted the chemoradiation‐caused enhancement of the BRD4‐IRF1 interaction (Figure 3F,G). IRF1 knockdown reversed PD‐L1 mRNA up‐regulation caused by the combination of radiation and cisplatin treatment (Figure 3H). Data mining in The Cancer Genome Atlas (TCGA) found that NSCLC patients with high BRD4 and high IRF1 mRNA levels had a higher PD‐L1 mRNA expression. While the PD‐L1 mRNA expression in NSCLC patients with low BRD4 and low IRF1 mRNA levels was relatively lower (Figure 3I,J). Of note, the CUT&Tag assay demonstrated that the BRD4‐IRF1 occupied the promoter of the PD‐L1 gene, and JQ1 could reverse both cisplatin‐enhanced and radiation‐enhanced enrichment of BRD4‐IRF1 on PD‐L1 promoter (Figure 3K,L). These suggest that JQ1 reversed chemoradiation‐induced PD‐L1 expression by regulating the BRD4‐IRF1 axis dependent PD‐L1 transcription in NSCLC.

Analysis of tissue microarray (TMA) from patients with NSCLC (Table S3) showed BRD4 protein expression is higher in tumour tissues compared with adjacent tissues (Figure 3M). We also observed a positive correlation between BRD4 protein expression and the percentage of PD‐L1 positive cells in the TMA (Figure 3N). By survival analysis in the TMA, the NSCLC patients with low BRD4 and low PD‐L1 had better survival outcomes than the other patients (Figure 3O). More specifically, the median overall survival time of the group with low BRD4 and low PD‐L1 was 52 months, and the other group was 33 months. However, the *p* value was .0862, which indicated the need for larger patient cohorts to validate the conclusion. Further analysis in the TCGA database, positive correlations between the BRD4‐IRF1‐PDL1 axis and the exhausted T cell signature score were observed in NSCLC, which suggested the important roles of this signalling axis in the immune tumour microenvironment (Figure S2A‐C).

### 3.4 Targeting BRD4 synergized with cisplatin and radiation in vivo

**FIGURE 4:**
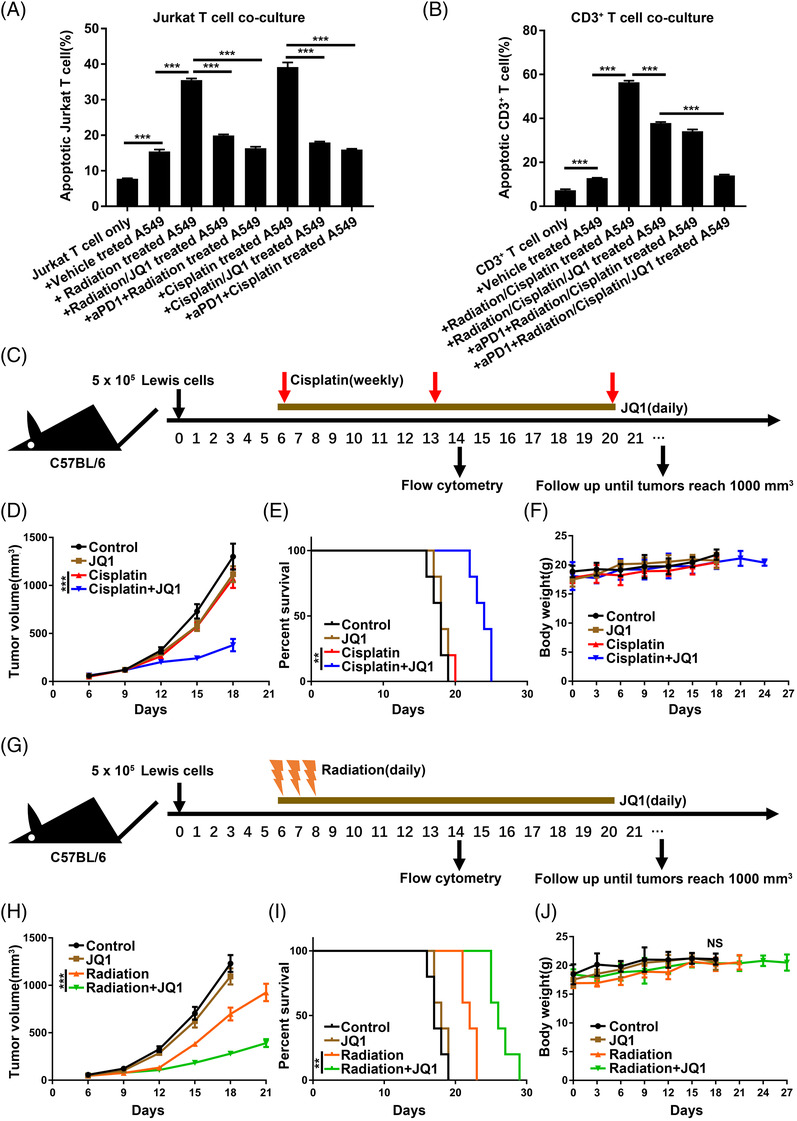
JQ1 enhanced the anti‐tumour effect of radiation and cisplatin in vivo. (A) Using the Annexin V‐FITC/PI assay, percentages of apoptotic Jurkat T cells were detected after co‐culturing with A549 cells with the indicated treatments (*n* = 3). (B) Apoptosis rates of CD3^+^ T cells were determined after co‐culturing with A549 cells with the indicated treatments (*n* = 3). (C) The schema for animal studies. Black arrows indicate the day of tumour implantation and analysis. The brown horizontal line represents JQ1 treatment. Red arrows indicate cisplatin treatment. Details of drug administration were described in the Methods. (D) Growth kinetics of implanted Lewis tumours treated as indicated. Error bars represent ± standard error of the mean (SEM) (*n* = 5). (E) Survival analysis and (F) body weight measurement for each of the indicated groups (*n* = 5). (G) The schema for animal studies. Black arrows indicate the day of tumour implantation and analysis. The brown horizontal line represents JQ1 treatment. The orange lightning bolt graphics represent X‐ray radiation. Details of drug administration and radiation were described in the Methods. (H) Growth kinetics of implanted Lewis tumours treated as indicated. Error bars represent ± SEM (*n* = 5). (I) Survival analysis and (J) body weight measurement for mice treated with the indicated regimens (*n* = 5)

Because tumour cell surface PD‐L1 level was up‐regulated in NSCLC cells following chemoradiotherapy and therapy‐induced PD‐L1 up‐regulation could potentially serve as a mechanism of resistance by inducing T cell exhaustion.^12^, ^13^, ^14^ Besides, recent reports showed that targeting the PD1/PD‐L1 signalling could boost the anti‐tumour immunity of radiation and cisplatin.^14^, ^17^ Hence, we then investigated whether the JQ1‐mediated reversion of chemoradiation‐stimulated tumour cell surface PD‐L1 up‐regulation in NSCLC would alleviate T cell apoptosis in co‐culture systems in vitro.^49^ The apoptosis level of Jurkat cells was increased when co‐cultured with the radiation‐treated and cisplatin‐treated A549 cells in contrast to that with the vehicle‐treated A549 cells or Jurkat cells alone group, and this increase was reversed by JQ1 and anti‐PD‐1 antibodies (Figure 4A). Similarly, A549 treated with the radiation and cisplatin combinatory therapy induced higher apoptosis levels in CD3^+^ T cells when compared with that in the vehicle‐treated A549 group; the induction was blocked by JQ1 and anti‐PD‐1 antibodies (Figure 4B). Therefore, we wonder whether BRD4 inhibition may boost anti‐tumour immunity and overcome resistance in vivo by suppressing chemoradiation‐induced PD‐L1 expression. The subcutaneous transplanted tumour model was used to test the hypothesis (Figure 4C). In immune‐competent C57BL/6 mice, JQ1 and cisplatin synergistically reduced tumour growth and prolonged mouse overall survival time without noted weight body loss or increased treatment‐related toxicities (Figure 4D‐F). However, we did not observe that JQ1 could significantly enhance the tumour‐killing effect of cisplatin in NSCLC in vitro or in immune‐deficient bagg albino (BALB) nude mice (Figure S3A‐C). These data indicated that the anti‐tumour effect of cisplatin and JQ1 combinatory treatment was dependent on intact T cell immunity. Similarly, JQ1 enhanced the tumour regression ability of radiation in C57BL/6 mice with intact immunity (Figure 4G–I) without observing significant bodyweight loss or other common side effects (Figure 4J).

### 3.5 JQ1 decreased chemoradiation‐induced PD‐L1 expression to augment CD8^+^ T cell‐dependent anti‐tumour immunity

**FIGURE 5:**
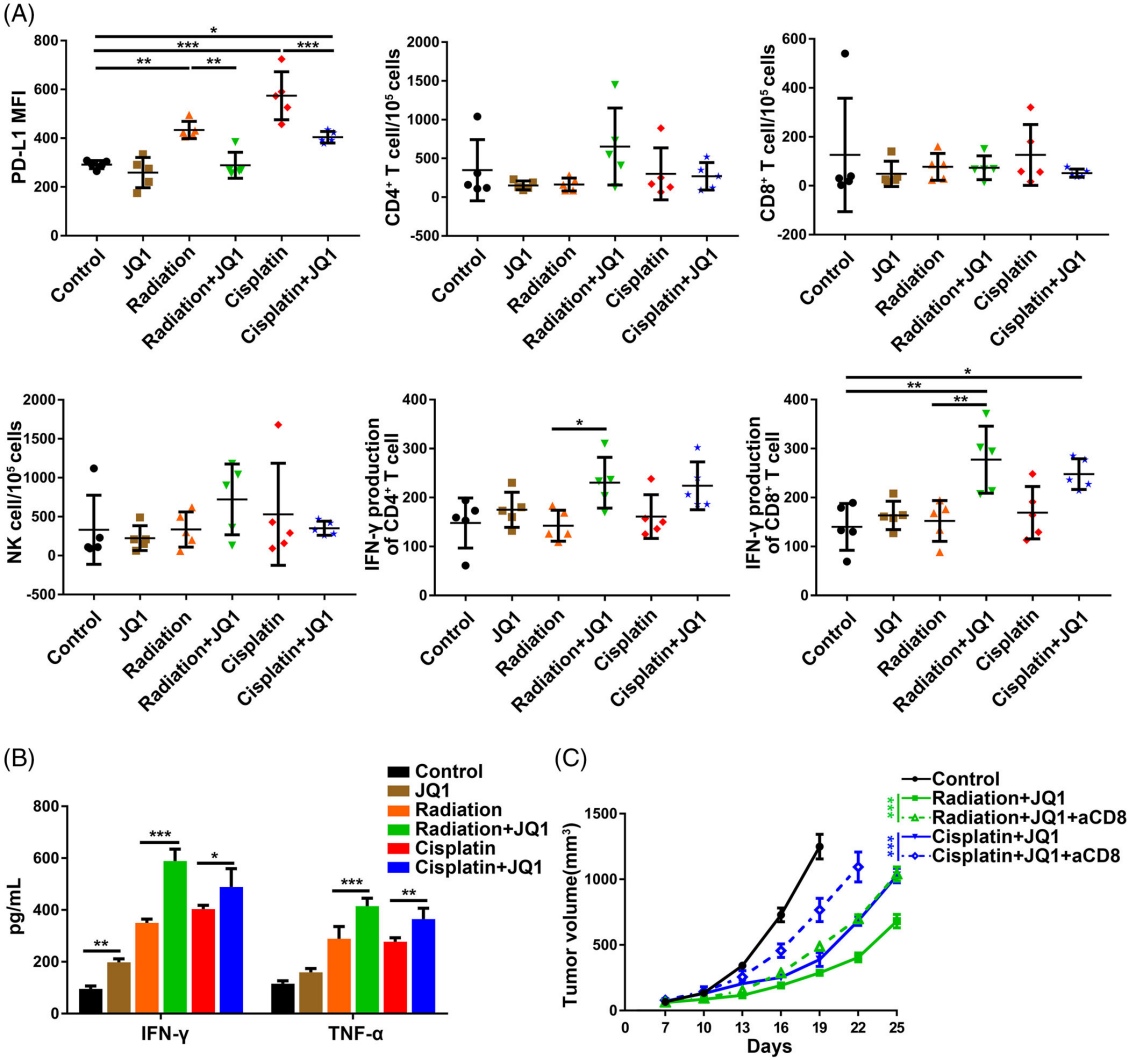
JQ1 enhanced anti‐tumour immunity of chemoradiotherapy dependent on CD8^+^ T cells. (A) Tumours were collected from each group of Lewis tumour‐bearing mice (*n* = 5) at day 14 after tumour implantation, 1 day after the second weekly dose of cisplatin, and 6 days after the third fraction of radiotherapy. Tumour cells and intratumoural immune cells were analyzed by flow cytometry. (B) Lewis tumour tissues were assessed for interferon gamma (IFN‐γ) and TNF‐α levels (*n* = 5). (C) Lewis‐bearing mice of the vehicle control, JQ1+radiation, JQ1+radiation anti‐CD8 antibody, JQ1+ CDDP and JQ1+ CDDP + anti‐CD8 antibody groups, were monitored for assessing tumour growth. Error bars represent ± SEM (*n* = 6)

Accumulating evidence showed that targeting PD‐L1 to overcome resistance to radiation and cisplatin by reprogramming tumour immune ecosystem.^17^, ^35^ Meanwhile, JQ1 is clinically safe and has been reported to enhance anti‐tumour immunity alone or in combination treatment mainly through regulating the activities of T cells and NK cells.^24^, ^25^, ^27^, ^28^ Because the T cell and NK cells are the most relevant to the anti‐tumour immunity of JQ1, radiation and cisplatin in preclinical models. To elucidate the synergistic mechanisms of JQ1 and chemoradiation combination treatment, we will focus our investigation on these immune cells. Consistent with results in vitro, flow cytometry analysis of tumours from different treated groups revealed that JQ1 suppressed the radiation‐induced and cisplatin‐induced tumour cell surface PD‐L1 up‐regulation in vivo (Figure 5A). There were no significant alterations in the number of CD4+ T cells, CD8^+^ T cells and NK cells in tumours received with JQ1 treatment combined with radiation and cisplatin. The combination of JQ1 and radiation treatment moderately increased IFN‐γ production of CD4+ T cells, while the same trend in the JQ1 and cisplatin combination treatment was observed without statistical significance. Notably, JQ1 combined with radiation and cisplatin treatment significantly enhanced the IFN‐γ production of tumour‐infiltrating CD8^+^ T cells in contrast to the treatment alone groups (Figure 5A). Additionally, JQ1 treatment with radiation and cisplatin synergistically increased the Tumour necrosis factor alpha (TNF‐α) and IFN‐γ levels, the major anti‐tumour cytokines secreted by cytotoxic T cells, in Lewis tumours (Figure 5B). We then depleted CD8^+^ T cells in a subgroup of Lewis‐bearing mice by anti‐mouse CD8α antibody and found that loss of CD8^+^ T cells largely impaired the combinatory regimens‐induced growth delay in vivo (Figure 5C). Collectively, these findings suggested that CD8^+^ T cells are necessary for the synergistic anti‐tumour effects of JQ1 with the combination of radiation and cisplatin.

### 3.6 JQ1 synergized with concurrent chemoradiotherapy and anti‐PD1 antibody without increasing toxicities

**FIGURE 6:**
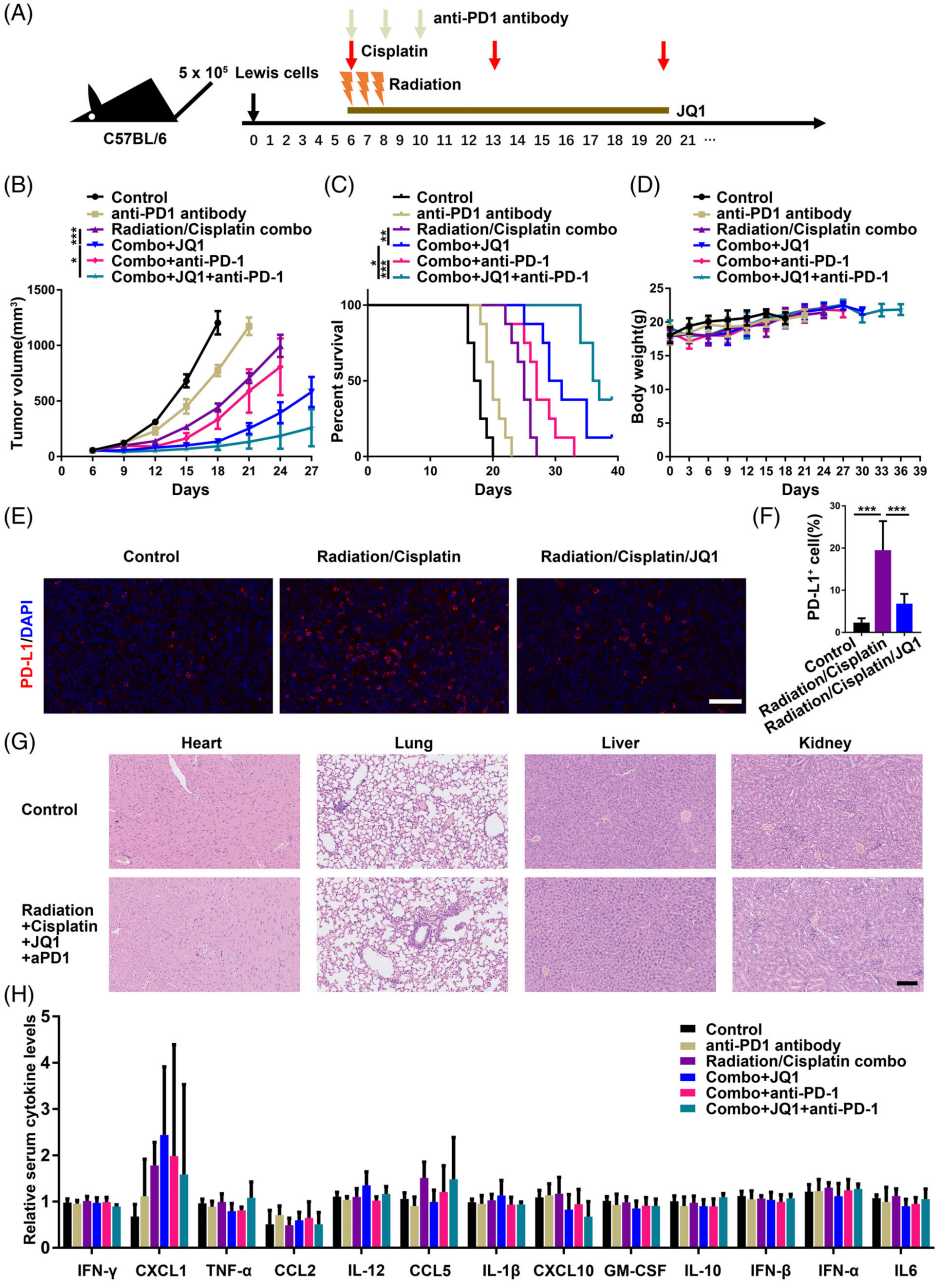
JQ1 synergized with concurrent chemoradiotherapy and anti‐programmed cell death‐1 (PD‐1) antibody without increasing toxicities. (A) The schema for animal studies. Black arrows indicate the day of tumour implantation. The brown horizontal line represents JQ1 treatment. Red arrows indicate cisplatin treatment. The orange lightning bolt graphics represent radiotherapy. Celadon green arrows symbolize anti‐PD‐1 therapy. Details of drug administration were described in the Methods. (B) Growth curves for implanted Lewis tumours treated as indicated (*n* = 8). (C) Survival analysis and (D) body weight measurement for each of the indicated groups (*n* = 8). (E) Immunofluorescence (IF) staining of PD ligand 1 (PD‐L1) in implanted Lewis tumours treated as indicated and counterstained with DAPI. Scale bar = 50 μm. (F) Quantitation of PD‐L1 positive cells in Lewis tumours after treatment as described (*n* = 5). (G) Hearts, lungs, livers and kidneys were harvested from Lewis‐bearing mice treated as indicated at the end of the experiments and prepared for hematoxylin‐eosin (H&E) analysis. Scale bar = 100 μm. (H) Serum was harvested from Lewis‐bearing mice treated as indicated and cryopreserved for cytokine analysis (*n* = 5). No significant changes were observed in the detected serum cytokines of the indicated groups

Next, we found that JQ1 combined with chemoradiation promoted stronger tumour regression and prolonged overall survival time of the Lewis‐bearing C57BL/6 mice without significant body weight loss in contrast to chemoradiotherapy (Figure 6A–D). IF staining of PD‐L1 showed that JQ1 restrained feedback PD‐L1 expression and decreased the number of PD‐L1 positive cells in vivo (Figure 6E,F). However, seven of eight of tumours ultimately resumed growth in the course of combinatory treatment. Thus, we investigated whether anti‐PD‐1 antibody may further improve the anti‐tumour effect of JQ1 and chemoradiotherapy in mice models. Results demonstrated that JQ1 combined chemoradiotherapy and anti‐PD‐1 antibody suppressed tumour growth and prolonged mouse survival in synergy (Figure 6A–C). Notably, three of eight tumours exhibited complete regression at day 60 in the JQ1 combined with chemoradiotherapy and anti‐PD‐1 treatment (data not shown), which suggested the improved capacity to eradicate existing tumours.

It is well‐known that cisplatin could cause clinically significant kidney toxicity in susceptible patients, and inflammatory cytokines may be involved in cisplatin‐induced nephrotoxicity,^50^ which could theoretically be worsened by immunotherapy‐related cytokine release syndrome (CRS). CRS is a severe complication with multiple organ damage and is associated with T‐cell activation and proliferation.^51^ Thoracic radiation‐induced damages include acute reactions like myocarditis and pneumonitis, which may develop into chronic fibrosis. Joint toxicities from radiotherapy and immune checkpoint blockade (ICB) are likely to increase the risk for cardiotoxicity^52^ and pulmonary toxicity.^53^ Special attention should be paid to the treatment‐related toxicities in the proposed combined therapy. First, there was no significant weight loss or any evidence of hearing impairment or sickness in mice treated with the JQ1 combined with chemoradiotherapy and anti‐PD‐1 antibody (Figure 6D). Second, none of the main organs (hearts, lungs, livers, kidneys) from mice in the group of JQ1 combined with chemoradiotherapy and anti‐PD‐1 antibody exhibited histologic signs of toxicity when compared with the control group treated with vehicle or untreated mice of the same age (Figure 6G). Third, a panel of serum cytokines was measured and showed that serum concentrations of IFN‐γ, interleukin 10 (IL‐10), TNF‐α, CXCL1, IL6, C‐C motif chemokine ligand 2 (CCL2), IFN‐α, CCL5, C‐X‐C motif chemokine ligand 10 (CXCL10), IFN‐β, granulocyte/macrophage colony stimulating factor (GM‐CSF), IL‐1β and IL‐12 all remained at basal or low levels across different groups at the end of treatment (Figure 6H). This suggests no marked CRS was observed in the combination therapy. Finally, we concluded that BRD4‐targeted therapy sensitized NSCLC to chemoradiation and anti‐PD‐1 antibody through a CD8+ T cell‐dependent mechanism without increasing the incidence and level of side effects.

## 4 DISSCUSSION

In this study, flow cytometry‐based drug library screenings and subsequent mechanistic studies led to the conclusion that BRD4 inhibitors, JQ1 and ARV771, are promising suppressors of chemoradiation‐induced PD‐L1 expression. Targeting BRD4 decreased treatment feedback PD‐L1 up‐regulation via blocking the recruitment of IRF1 to PD‐L1 promoter and sensitized NSCLC cells to chemoradiotherapy and anti‐PD‐1 antibody through a cytotoxic T cell‐dependent manner without increasing toxicities.

Cisplatin‐based chemotherapy and radiotherapy have been extensively used in the management of patients with NSCLC. Moreover, concurrent chemoradiotherapy is the standard treatment for stage III NSCLC.^3^, ^54^ It has been suggested that radiation and cisplatin could modify host immunity to synergize with immunotherapy in several tumour types by enhancing tumour immunogenicity.^35^ Chemoresistance and radioresistance are clinical challenges that weaken treatment effects and eventually cause cancer treatment failure. However, researchers recently have found activated PD‐1/PD‐L1 signalling in chemoresistant^55^ or radioresistant^15^ tumour cells, which potentially promotes tumour immune evasion by inhibiting tumour‐specific T cell activity. Furthermore, cisplatin and radiation up‐regulated inducible PD‐L1 expression by multiple mechanisms including ATR/ATM/JAK/STAT/IRF1 signalling,^13^, ^17^ PI3K/AKT axis,^15^ cGAS/STING pathways,^14^ etc. We also demonstrated the additional up‐regulation of PD‐L1 expression by the combination of radiation and cisplatin. Therefore, therapy‐induced PD‐L1 expression on tumour surfaces justified the combinatory treatment of chemoradiotherapy and targeting PD‐L1/PD‐1 therapy.

However, screen projects to explore the unique regulators of chemoradiation‐induced PD‐L1 upregulation are rare. Herein, we performed the first flow cytometry‐based screening experiment to identify unique drugs, which can regulate both radiation‐induced and cisplatin‐induced PD‐L1 upregulation in NSCLC, out of a customed drug array with more than two hundred compounds that mainly are composed of kinase inhibitors and epigenetic inhibitors. By overlapping the candidates from the radiation screens and the cisplatin screens in two NSCLC cells, we found BRD4 inhibitor JQ1, PI3KCA inhibitor Alpelisib, and BRD9 inhibitor I‐BRD9 that satisfy the criteria. Supporting the reliability of results in the screening assay, it is reported that PI3KCA inhibition reduced PD‐L1 expression in radioresistant head and neck cancer.^40^ Because JQ1‐based treatment could reprogram the tumour immune ecosystem via multiple mechanisms,^22^, ^24^, ^26^, ^27^, ^28^, ^29^ we further evaluate the role of JQ1 in chemoradiation‐stimulated PD‐L1 expression. Given that IFN‐γ secreted by immune cells including activated CD8^+^ T cells stimulated PD‐L1 expression, we confirmed that JQ1 inhibited IFN‐γ‐induced PD‐L1 up‐regulation. BRD4 depletion by siRNAs and a BRD4‐target degrader ARV771 suppressed radiation‐induced and cisplatin‐induced PD‐L1 up‐regulation in NSCLC. IRF1 is a well‐known transcription factor to regulate tumour cell PD‐L1 expression by binding to CD274 promoter,^13^, ^45^, ^46^ and BRD4 is another key mediator of both tumour cell constitutive and IFN‐γ‐stimulated CD274 transcription.^24^, ^47^ BRD4 could interact with IRF1 to facilitated MLKL transcription.^48^ Our results showed that BRD4 could recruit IRF1 to the promoter of PD‐L1 to increase PD‐L1 transcription. Chemoradiation increased the binding of BRD4‐IRF1 to PD‐L1 promoter, and JQ1 blocked the increase. Data analysis in patients with NSCLC demonstrated that there were significant positive correlations among the BRD4‐IRF1‐PD‐L1 signalling and exhausted T cell status by using the TCGA database and a TMA cohort. These mechanistic findings showed that JQ1 reversed chemoradiation‐induced PD‐L1 expression by impeding the BRD4‐IRF1 axis dependent PD‐L1 transcription in NSCLC. However, to validate the combination of BRD4 and PD‐L1 expression as the prognostic factors in NSCLC may require larger cohorts.

PD‐L1 has been shown to mediate tumour resistance to radiotherapy,^15^ chemotherapy^55^ and immunotherapy.^56^ We found that JQ1 could limit therapy‐induced PD‐L1 up‐regulation in vivo and enhanced the anti‐tumour efficacy of radiation and cisplatin in immune‐intact tumour‐bearing animal models. The JQ1‐based combination therapy enhanced CD8^+^ T cell activity, and the synergistic anti‐tumour effect of JQ1 and chemoradiation was significantly diminished by CD8^+^ T cell depletion. Adding JQ1 and the anti‐PD‐1 antibody could further enhance the tumour regression activity of chemoradiation. Thus, these data suggest that JQ1 overcome resistance to chemoradiotherapy through a CD8^+^ T cell‐dependent mechanism by inhibiting treatment‐induced feedback PD‐L1 up‐regulation in vivo. ICB has been reported to lead to durable tumour regression, but the response rate is relatively low.^57^ Recent studies showed that radiotherapy and cisplatin‐based chemotherapy boosted anti‐tumour effects of ICB through multiple mechanisms including enhancing tumour immunogenicity.^35^ Previous studies showed that JQ1 synergizes with anti‐PD‐1/PD‐L1 antibodies in lymphomas^23^ and Kras‐mutant NSCLC.^29^ However, JQ1 could only suppress chemoradiation‐induced PD‐L1 up‐regulation to the level before chemoradiotherapy; thus the residual constitutive PD‐L1‐mediated negative signals would continue to cause T cell exhaustion.^58^, ^59^ In this study, anti‐PD‐1 antibody decreased T cell apoptosis level in the co‐culture of CD3+ T cells and NSCLC cells treated with JQ1 and chemoradiotherapy while adding anti‐PD‐1 antibodies to the combination of JQ1 and chemoradiotherapy might further restrain the negative regulatory effect of PD‐1/PD‐L1 signalling on the anti‐tumour activity of T cells. Hence, JQ1 may increase the tumour response rate to chemoradiotherapy combined with ICB and improve tumour control.

We also attempted to determine whether the combination treatment would add to the side toxicities, which may be a main barrier for the clinical translation. Importantly, there were no significant body weight loss and other common toxic effects in animal experiments. We did not find detectable histological changes in the heart, lung, liver or kidney tissues in the combinatory treatment group compared with the control group. Besides, a panel of serum cytokine measurements showed no significantly increased cytokines across different groups, indicating no marked CRS in JQ1 combined with chemoradiotherapy and anti‐PD‐1 therapy.

In summary, we found that targeting BRD4 suppressed radiation‐induced and cisplatin‐induced PD‐L1 up‐regulation through disrupting the recruitment of IRF1 to PD‐L1 promoter and enhance the anti‐tumour immunity of chemoradiation in a CD8^+^ T cell‐dependent manner in NSCLC without significantly increased treatment‐related toxicities. This study provides pre‐clinical evidence for targeting BRD4 to augment anti‐tumour immunity of chemoradiotherapy and anti‐PD‐1 in NSCLC.

## CONFLICT OF INTEREST

The authors declare no potential conflict of interest.

## Supporting information



Table S1 Results related to drug screens.

Table S2 Gene lists related to the JQ1 signatures.

Table S3 Association between the clinicopathologic variables and expression of BRD4 in NSCLC tissue microarray.

Figure S1 JQ1 attenuated chemoradiotherapy‐induced transcription of PD‐L1 and increased MHC‐1 expression.

Supplementary Materials

## Data Availability

The data that supports the findings of this study are available in the supplementary material of this article.
