# Supplementary material for: BRD4‐IRF1 axis regulates chemoradiotherapy‐induced PD‐L1 expression and immune evasion in non‐small cell lung cancer
Source: Clin Transl Med. 2022 Jan 26;12(1):e718. doi: 10.1002/ctm2.718 (PMC8792480; doi:10.1002/ctm2.718)
Supplement: Supplementary file 3 — Table S3 Association between the clinicopathologic variables and expression of BRD4 in NSCLC tissue microarray. [file CTM2-12-e718-s005.docx]

**Table S3 Association between the clinicopathologic variables and expression of BRD4 in NSCLC tissue microarray.**

| **Features** | **BRD4 expression** | | ***P* value** |
| --- | --- | --- | --- |
|  | **low** | **high** |  |
| **Total** | 43 | 55 |  |
| **Mean age(years)** | 58.4 | 61.1 |  |
| **Age (years)** |  |  |  |
| ≤60 | 25 | 26 | 0.285 |
| >60 | 18 | 29 |  |
| **Gender** |  |  |  |
| Male | 21 | 34 | 0.199 |
| Female | 22 | 21 |  |
| **Stage** |  |  |  |
| I | 16 | 17 | 0.567 |
| II | 11 | 14 |  |
| III | 15 | 24 |  |
| IV | 1 | 0 |  |
| **T stage** |  |  |  |
| 1 | 10 | 10 | 0.542 |
| 2 | 23 | 28 |  |
| 3 | 9 | 12 |  |
| 4 | 1 | 5 |  |
| **N stage** |  |  |  |
| 0 | 21 | 24 | 0.826 |
| 1 | 12 | 14 |  |
| 2 | 5 | 10 |  |
| 3 | 5 | 7 |  |
| **M stage** |  |  |  |
| 0 | 42 | 54 | 0.260 |
| 1 | 1 | 0 |  |
| **No. of death** | 29 | 38 |  |

The *P* value was calculated with the Pearson χ2 test.
