# Supplementary material for: BRD4‐IRF1 axis regulates chemoradiotherapy‐induced PD‐L1 expression and immune evasion in non‐small cell lung cancer
Source: Clin Transl Med. 2022 Jan 26;12(1):e718. doi: 10.1002/ctm2.718 (PMC8792480; doi:10.1002/ctm2.718)
Supplement: Supplementary file 4 — Figure S1 JQ1 attenuated chemoradiotherapy‐induced transcription of PD‐L1 and increased MHC‐1 expression. [file CTM2-12-e718-s004.docx]

**JQ1 inhibits chemoradiotherapy-induced PD-L1 expression via the BRD4-IRF1 axis and augments antitumor immunity in NSCLC**


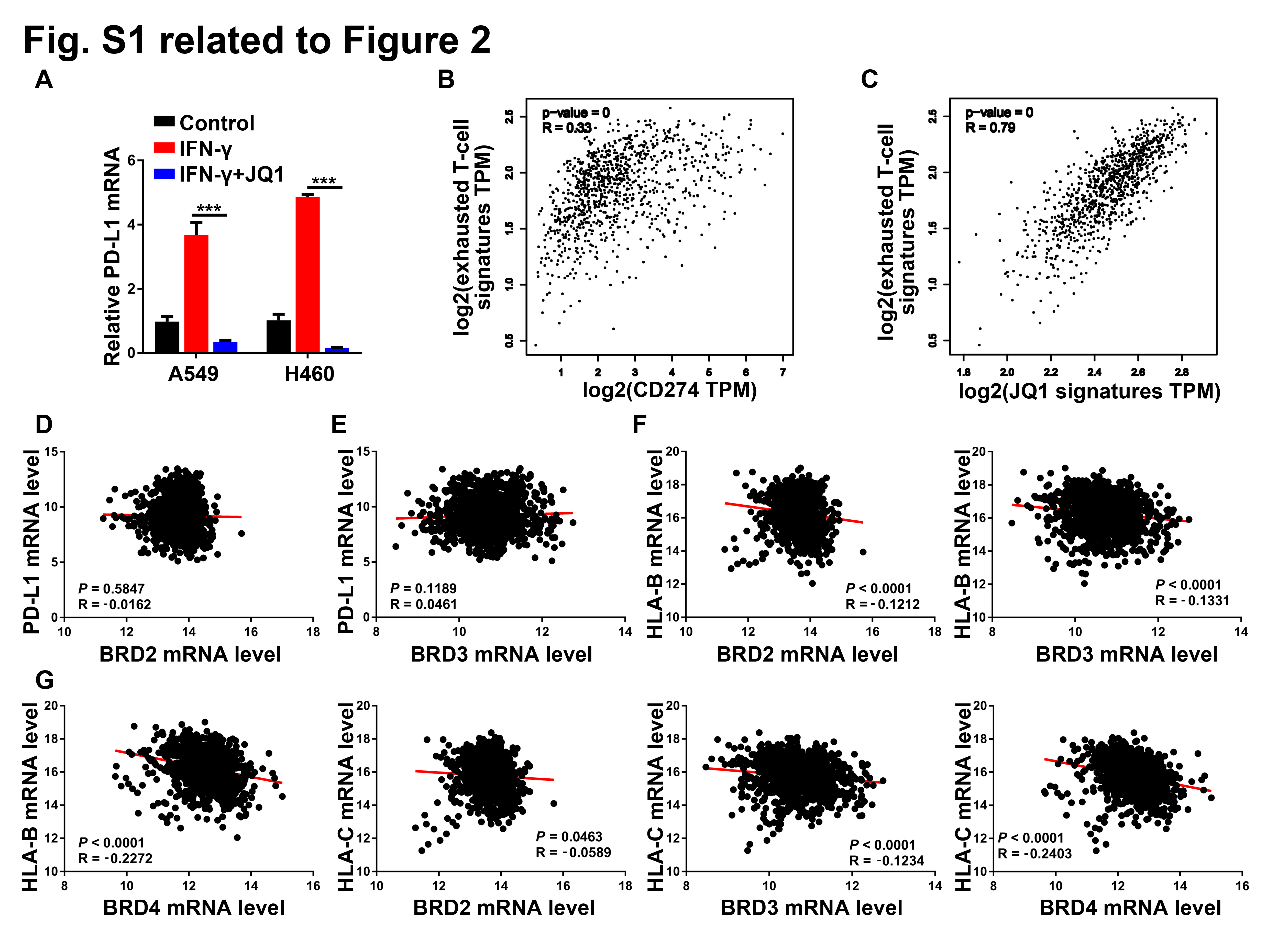


### Fig. S1 related to Fig. 2 JQ1 attenuated chemoradiotherapy-induced transcription of PD-L1 and increased MHC-1 expression

(A) Changes of PD-L1 mRNA in NSCLC cell lines stimulated with IFN-γ in the presence or absence of JQ1 (1 μM) for 36 h. Results are normalized to GAPDH (n = 3). (B) The correlation of the exhausted T cell signature and CD274 mRNA expression in NSCLC by analysis of TCGA data. (C) The correlation of the exhausted T cell signature and the JQ1 signature in NSCLC by analysis of TCGA data. (D) and (E) The correlation of PD-L1 mRNA expression and BRD2/3 in NSCLC by analysis of TCGA data. (F) and (G) The correlation of HLA-B/C mRNA expression and BRD2/3/4 in NSCLC by analysis of TCGA data.


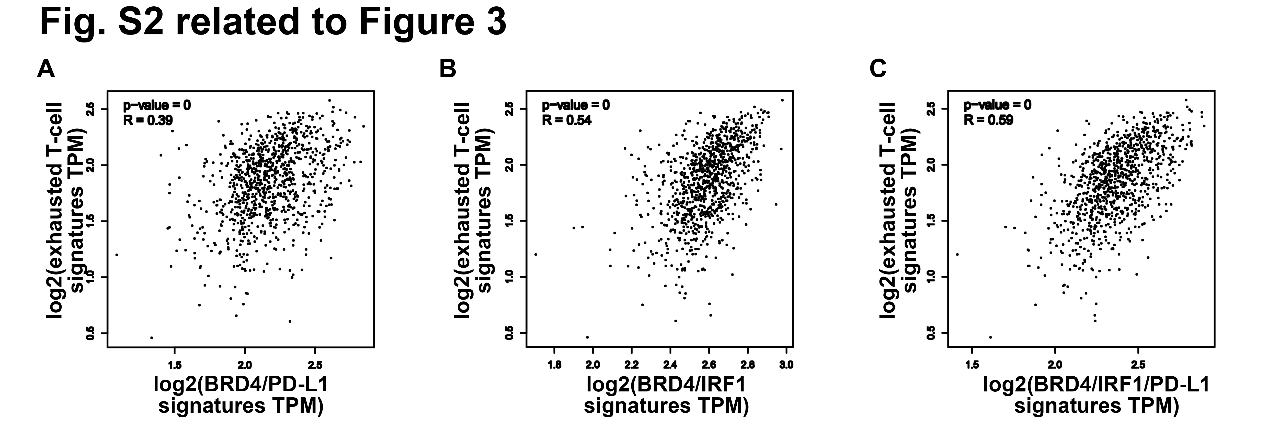


### Fig. S2 related to Fig.3 The BRD4-IRF1 axis is required for chemoradiotherapy-mediated PD-L1 upregulation

(A) The correlation of the exhausted T cell signature and the BRD4/PD-L1 signature in NSCLC by analysis of TCGA data. (B) The correlation of the exhausted T cell signature and the BRD4/IRF1 signature in NSCLC by analysis of TCGA data. (C) The correlation of the exhausted T cell signature and the BRD4/IRF1/PD-L1 signature in NSCLC by analysis of TCGA data.


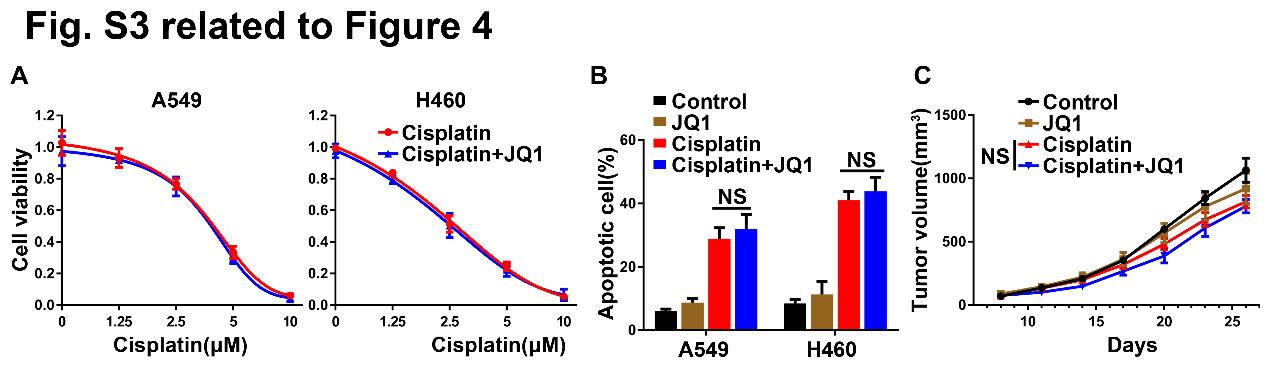


### Fig. S3 related to Fig.4 JQ1 enhanced the anti-tumor effect of radiation and cisplatin in vivo.

(A) Cell viability of NSCLC cells treated with the indicated concentrations of cisplatin in the presence or absence of JQ1(1 μM) for 48h. (B) The apoptosis index of NSCLC cells treated with cisplatin (4 μM) and JQ1(1 μM) for 48h. (C) H460-bearing BALB nude mice were treated with cisplatin and JQ1 and the tumor volumes were monitored (n = 5).

### Fig. S4 Graphical Abstract.


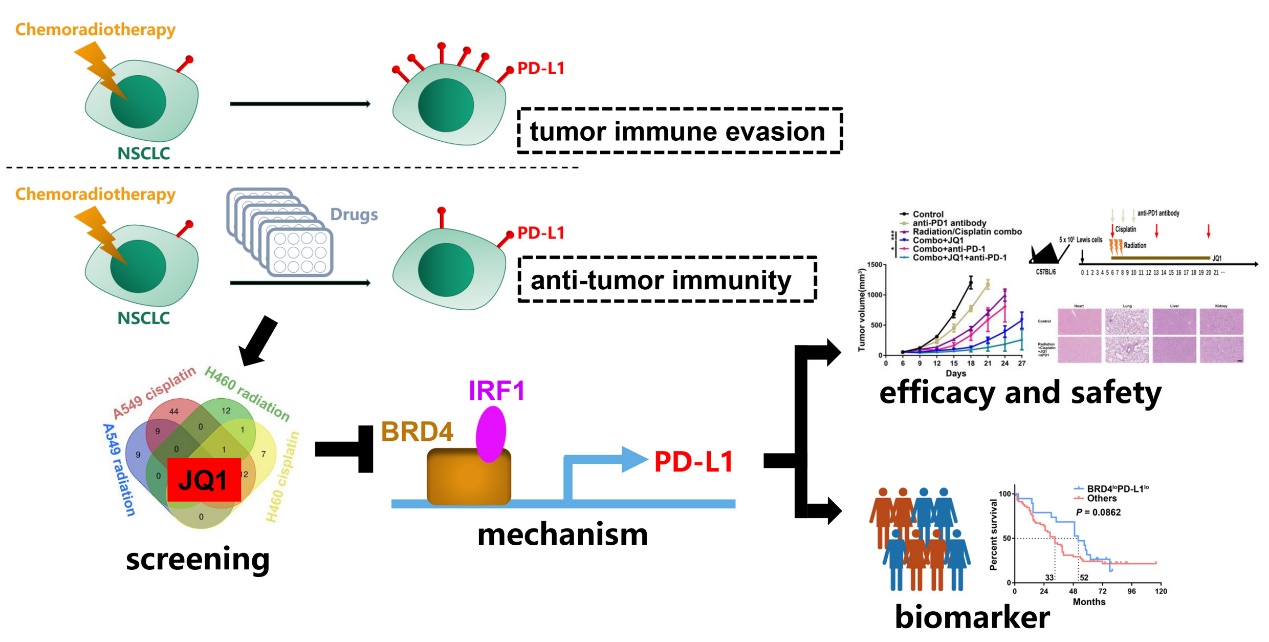


Drug screening experiments show that JQ1 is an inhibitor of cisplatin-induced and radiation-induced PD-L1 upregulation in NSCLC. Chemoradiotherapy can upregulate PD-L1 expression by recruiting BRD4-IRF1 to the PD-L1 promoter region. BRD4 inhibitor JQ1 can sensitize NSCLC to chemoradiotherapy and anti-PD-1 antibodies via CD8+ T cells without increasing toxicities.
