# Supplementary material for: BRD4‐IRF1 axis regulates chemoradiotherapy‐induced PD‐L1 expression and immune evasion in non‐small cell lung cancer
Source: Clin Transl Med. 2022 Jan 26;12(1):e718. doi: 10.1002/ctm2.718 (PMC8792480; doi:10.1002/ctm2.718)
Supplement: Supplementary file 5 — Supplementary Materials [file CTM2-12-e718-s002.docx]

**JQ1 inhibits chemoradiotherapy-induced PD-L1 expression via the BRD4-IRF1 axis and augments antitumor immunity in NSCLC**

**Cell lines,** **reagents, and radiation**

Cells (A549, H460, H1299, H1975, H292, Lewis) were obtained from American Type Culture Collection (ATCC) and cultured in recommended medium supplemented with 10% heat-inactivated fetal bovine serum and 1% penicillin/streptomycin, at 37 °C in a humidified incubator containing 5% CO_2_. Cells were authenticated and confirmed mycoplasma free. A customed drug library, JQ1, and human anti-PD-1 were purchased from Selleck. Cisplatin, ARV-771, and APTO253 were purchased from MedChemExpress. Recombinant human IFN-γ was purchased from PeproTech. The reagents were resuspended and stored according to instructions. Tumor cells and animal xenografts were exposed to the indicated doses of radiation respectively by 6 MV X rays at a dose rate of 600 MU/min using an X-ray irradiator (Varian).

## Real-time quantitative PCR (RT-qPCR)

RNA was extracted with TRIzol (Invitrogen) and quantitated using the NanoDrop1000 (Thermo Fisher Scientific). The purified RNA was reverse transcribed into cDNA with RT Master Mix (TaKaRa). PCR reaction was performed with specific primers by using SYBR Green real-time PCR kit (TaKaRa) in a StepOne Plus PCR System (Applied Biosystems). The relative expressions of target genes were determined by using the comparative threshold cycle method and GAPDH in each sample was used as an internal control. The primer sequences were designed according to published study[1] and the following primers were used, human *CD274*, forward: 5′-GGAGATTAGATCCTGAGGAAAACCA-3′, reverse: 5′-AACGGAAGATGAATGTCAGTGCTA-3′; human *GAPDH*, forward: 5′-CTCCTCTGACTTCAACAGCGA-3′, reverse: 5′-CCAAATTCGTTGTCATACCAGGA-3′; human *HLA-A*, forward: 5′-AGATACACCTGCCATGTGCAGC-3′, reverse: 5′-GATCACAGCTCCAAGGAGAACC-3′; human *HLA-B*, forward: 5′-CTGCTGTGATGTGTAGGAGGAAG-3′, reverse: 5′-GCTGTGAGAGACACATCAGAGC-3′; human *HLA-C*, forward: 5′-GGAGACACAGAAGTACAAGCGC-3′, reverse: 5′-ACATCCTCTGGAGGGTGTGAGA-3′.

## Cell viability assay and apoptosis detection

Cells were cultured and treated as described, and then cell viability was detected by CCK-8 kit (Vazyme Biotech) according to the manufacturer’s instructions. Single-cell suspensions were harvested and the apoptosis index was detected using an Annexin V/propidium iodide (PI) staining detection kit (Vazyme Biotech) in a CytoFlex S flow cytometer (Beckman Coulter) following the manufacturer’s instructions and analyzed with FlowJo (v10).

## Gene silencing

All siRNAs were transfected into cells using Lipofectamine RNAimax (Invitrogen) following the manufacturer’s protocol and validated at 48 h after transfection by Western blot. The used sequences of the siRNAs were designed according to published studies[2-4].

## Western blot and immunoprecipitation (IP)

Cells were homogenized in NETN lysis buffer supplemented with protease inhibitor and phosphatase inhibitor for total protein extracts. The lysates were denatured at 100°C for 10 min and subjected to SDS-PAGE electrophoresis. After transferring proteins onto PVDF membranes, primary antibodies and appropriate HRP-conjugated secondary antibodies were used for staining and detection. For the co-IP assay, the lysates were first precleared with protein A/G beads, then incubated with appropriate antibodies or isotype control IgG on a rotator overnight at 4°C and then with protein A/G-agarose at 4°C for 1 hour. The precipitates were washed 5 times with lysis buffer and subjected to immunoblotting. The following antibodies were used: PD-L1(#13684, Cell Signaling Technology), βactin (AC026, ABclonal), BRD2(A16241, ABclonal), BRD3(A2277, ABclonal), BRD4(ab128874, Abcam; sc-518021, Santa Cruz Biotechnology), IRF1(#8478, Cell Signaling Technology; sc-74530, Santa Cruz Biotechnology).

## Immunofluorescence (IF) and immunohistochemistry (IHC), and hematoxylin eosin (H&E) staining

Cells were cultured onto slides and treated as indicated, and then fixed with 4% paraformaldehyde and blocked with 5% BSA for 1 h at room temperature. Primary antibodies and appropriate Alexa Fluor 594 dye-conjugated secondary antibodies were used for detection and visualization. Paraffin-embedded tissue sections were processed following standard protocols and stained with appropriate antibodies for IF and IHC tests. Tissue microarrays of patients with NSCLC were obtained from Shanghai Outdo Biotech (HLugA180Su07). The IHC analysis gives a score of 0 to 3 that measures the intensity of protein stain and 0 to 4 that measures the percentage of positive cells in tissue samples. “BRD4 high” sample is defined as the BRD4 score (multiplying intensity by percentage) is more than 3. “PD-L1 high” sample is defined as the percentage is more than 10%. The following antibodies were used: anti-PD-L1 (ab205921, Abcam; 66248-1-Ig, Proteintech), BRD4 (ab128874, Abcam). For histological structure analysis, HE staining in paraffin-embedded tissue was conducted by using Hematoxylin and Eosin Staining Kit (Abcam) following the manufacture’s protocol.

## RNA-seq and database analysis

Gene expression analysis was conducted by RNA-seq following standard protocols for the conditions described in the relevant figures. Differential expression genes (DEGs) were defined as |fold change| ≥ 2 and *P* value ≤ 0.001 (Table S2). The transcriptomic data of Lung adenocarcinoma (LUAD) and Lung squamous cell carcinoma (LUSC) in The Cancer Genome Atlas (TCGA) were normalized and reanalyzed and the median values were used as the cutoff point to classify patients. The interferon gamma (IFN-γ) response gene set and the adaptive immune system gene data set were downloaded from The Molecular Signatures Database (MSigDB). The immune gene signatures including the exhausted T-cell signature (*HAVCR2, TIGIT, LAG3, PDCD1, CXCL13, LAYN*), the JQ1 signature (*VCAM1, SLAMF7, CD274, MYD88, PTPN6, HERC6, PSMB9, TAPBP*), and the BRD4-IRF1-PD-L1 axis were analyzed in NSCLC (including LUAD and LUSC) by GEPIA[5].

**Cytokine assays**

Tissues were weighed and homogenized for IFN-γ and TNF-α detection by using Quantikine ELISA (R&D Systems) following the manufacturer’s instructions. The serum was cryopreserved and cytokines were measured with the Multi-Analyte Flow Assay Kit (Biolegend) according to the manufacturer’s instructions by flow cytometry.

## In vitro co-culture assay

Human peripheral blood mononuclear cells (PBMCs) were isolated by density centrifugation with Ficoll-Paque. CD3^+^ primary human T cells were sorted from PBMC using the T cell isolation kit (Stemcell Technologies). T cells were activated with the CD3/CD28 T cell activator (Stemcell Technologies) for 24 h or 48 h[6]. Tumor cells were treated with radiation, cisplatin, JQ1, anti-PD-1 antibodies (aPD1), and the combinations for 24 h or 48 h before co-culturing with activated Jurkat T cells or CD3^+^ T cells[7]. T cells were subjected to apoptosis detection by Annexin V-FITC/PI assay.

# Reference

1 Sato H, Niimi A, Yasuhara T, Permata TBM, Hagiwara Y, Isono M *et al*. DNA double-strand break repair pathway regulates PD-L1 expression in cancer cells. *Nature communications* 2017; 8: 1751.

2 Tian CQ, Chen L, Chen HD, Huan XJ, Hu JP, Shen JK *et al*. Inhibition of the BET family reduces its new target gene IDO1 expression and the production of L-kynurenine. *Cell death & disease* 2019; 10: 557.

3 Lai Q, Wang H, Li A, Xu Y, Tang L, Chen Q *et al*. Decitibine improve the efficiency of anti-PD-1 therapy via activating the response to IFN/PD-L1 signal of lung cancer cells. *Oncogene* 2018; 37: 2302-2312.

4 Xiao G, Jin LL, Liu CQ, Wang YC, Meng YM, Zhou ZG *et al*. EZH2 negatively regulates PD-L1 expression in hepatocellular carcinoma. *Journal for immunotherapy of cancer* 2019; 7: 300.

5 Tang Z, Li C, Kang B, Gao G, Li C, Zhang Z. GEPIA: a web server for cancer and normal gene expression profiling and interactive analyses. *Nucleic acids research* 2017; 45: W98-W102.

6 Cerezo M, Guemiri R, Druillennec S, Girault I, Malka-Mahieu H, Shen S *et al*. Translational control of tumor immune escape via the eIF4F-STAT1-PD-L1 axis in melanoma. *Nature medicine* 2018.

7 Zhang Q, Zhang Y, Chen Y, Qian J, Zhang X, Yu K. A Novel mTORC1/2 Inhibitor (MTI-31) Inhibits Tumor Growth, Epithelial-Mesenchymal Transition, Metastases, and Improves Antitumor Immunity in Preclinical Models of Lung Cancer. *Clinical cancer research : an official journal of the American Association for Cancer Research* 2019; 25: 3630-3642.
